# Mesomorphic and DFT study of new difluoro substituted Schiff base liquid crystals

**DOI:** 10.1038/s41598-025-97375-8

**Published:** 2025-05-03

**Authors:** Manav Jeetendra Shirodkar, K. Subrahmanya Bhat, Debanjan Bhattacharjee, M. K. Sonali, M. G. Mahesha, Poornima Bhagavath

**Affiliations:** 1 Department of Chemistry, Manipal Institute of Technology, Manipal Academy of Higher Education, Manipal, Karnataka 576 104 India; 2 Department of Physics, Manipal University Jaipur, Jaipur, India; 3 Department of Physics, Manipal Institute of Technology, Manipal Academy of Higher Education, Manipal, Karnataka 576 104 India

**Keywords:** Liquid crystals, Nematic, Smectic A, Fluoro substituents, Mesomorphic, Chemistry, Materials science

## Abstract

New liquid crystal (LC) molecules with Schiff base linkage and fluoro substituents are synthesized and characterized. It is anticipated that the presence of highly electronegative and small sized fluorine atoms influences the liquid crystal’s mesomorphic behavior, while the Schiff base structure enhances polarizability and molecular interactions. The new liquid crystals are characterized by ^1^H NMR spectroscopy, Fourier-transform infrared spectroscopy (FTIR), polarized optical microscopy (POM) and differential scanning calorimetry (DSC). Nonlinear optical properties (NLO) are studied through DFT measurements. The findings of the DSC and POM investigations show that the molecule’s mesophase behavior is significantly influenced by the terminal alkoxy chain length. Investigating the molecule’s nonlinear optical characteristics also produced encouraging findings for its possible use in photonic devices. The ability to refine the mesomorphic behavior by varying the alkoxy chain length provides a notable advantage in designing materials with specific properties for optical switching and display technologies.

## Introduction

Liquid crystals (LCs) are unique state of matter that has properties between those of liquids and solid crystals. This makes them crucial materials in a wide range of applications, from sensors to display technologies. The different molecular arrangements in LCs produce phases with differing levels of symmetry and order between the isotropic and crystalline phases. In terms of technology, thermotropic LCs are particularly significant for displays and other applications^1^. The molecular structure of rod-shaped liquid crystals controls their mesomorphic characteristics, and even little changes in geometry can have a major impact on mesomorphic behavior^2^. The formation, kind of mesophase, and thermal stability of the compounds under investigation are significantly influenced by the terminal arms and connecting units. Terminal groups significantly affect thermal stability by influencing the polarity and molecular polarizability. It is also feasible to modify the liquid crystal’s phase transition temperature and electro-optical sensitivity by modifying the linking group^3^. A sufficient length of the flexible chain, terminal groups, and the mesogenic core are three factors that affect the molecular structure of thermotropic LCs molecules^4^. In recent decades, there has been a lot of focus on creating fluorinated LCs^5–13^. The fluorine atom possesses various unique features, and its incorporation into organic compounds results in substantial enhancements or modifications of their physical characteristics^14^. The most common methods for joining units at the central portions of many liquid crystalline molecular systems are ester, Schiff base, or chalcone groups^15–17^. To gain a better understanding of the connection between the molecular geometry of the mesogens and their mesomorphic features, several two- and three-ring compounds derived from Schiff base/ester liquid crystals are being studied for their optical properties^18^. It is observed that phenyl rings and other rigid structures benefit from the addition of –HC=N connections, which maintain structural linearity and increase mesophase stability. Schiff base linking groups offer a number of advantages when used as a connecting group within liquid crystal molecules. They can be employed in a range of mesogenic topologies and are easy to synthesize. Additionally, the imine bond is quite strong, which helps to maintain the liquid crystal phase’s stability throughout a wide temperature range.

It is a well-known observation that, Schiff base LCs possessing varied substituents such as –F, –Cl, –CN, –CH_3_, –OCH_3_ in the terminal and/or the lateral positions influence the liquid crystalline property to a great extent. Benzoic acids react with alcohols/phenols to produce esters, and these reactions are largely influenced by the substituents of the benzoic acids. The alkyl and alkyloxy substituents are electron donating in nature and influence the acidity of the benzoic acids while halo substituents influence the acidity through their electron withdrawing behaviour. Liquid crystals imbibing fluorine atoms in their molecular structure is well appreciated by various research groups since decades^19–22^ as these mesomorphic compounds are found to possess reduced viscosity and adjustable dielectric anisotropy. The versatile nature of the fluorine atom in possessing high electronegativity with small size are responsible for the liquid crystal molecules in possessing these outstanding features. New fluorinated liquid crystals have been synthesized by Chen et.al^23^ by introducing cyclohexyl and bicyclohexyl moieties in the rigid cores of the liquid crystalline materials in anticipation of obtaining high birefringence materials. Series of mono, di and tri fluoro substituted liquid crystals were synthesized. It is observed that with the increase in the width of the mono, di and tri substituted compounds, the increase in the intermolecular separation weakened the lateral molecular attractive forces and at the same time the increased molecular polarity is found to enhance the terminal molecular attractive force. Various Schiff bases were synthesized by Sie-Tiong Ha et. al^24^ by considering ortho hydroxy substituted aldehydes with different para substituents like –F, –Cl, –Br, –OCH_3_, –CH_3_ and –C_2_H_5_ on the aniline counterpart. Amongst these compounds, the clearing temperature for the compound possessing fluorine in the aniline fragment is found to be much lower than the chloro and bromo containing compounds. This is attributed to the decrease in the degree of molecular order in fluorine containing compound due to the most electronegative nature of fluorine. Thermal data indicated that the clearing temperature of fluoro substituted compound decreased in comparison with chloro and bromo substituted compounds. Fluorine substituted liquid crystals presented by our group^25^ earlier is found to exhibit smectic mesomorphism with para fluoro substituted liquid crystal series exhibiting highest mesomorphism and meta fluoro substituted liquid crystals exhibited lowest mesomorphic thermal stability. These observations are attributed to the enhanced longitudinal dipole moment of the molecules with fluorine as the terminal substitution and increase in the breadth of the molecule with the fluorine in the lateral position.

In the present work, we report the synthesis of two new LC molecules, 4-((2′,4′-diflourophenylimino)methyl)phenyl-4′′-pentyloxybenzoate (5OFB) and 4-((2′,4′-diflourophenylimino)methyl)phenyl-4″-dodecyloxybenzoate (12OFB) and study the effect of the alkoxy chain length and the polarity of the fluoro substituents on the mesomorphic properties. The chemical, phase and thermal characterization are discussed and the nonlinear optical properties of 5OFB carried out through DFT studies is described. A wide range of nematic mesophase is observed in the shorter chain length compounds while the higher chain length compounds exhibited a wide range of smectic mesomorphism.

## Experimental

### Materials and methods

The chemicals and solvents used for the synthesis of 5OFB and 12OFB are procured from Sigma Aldrich, and Spectrochem Chemicals Pvt. Ltd. The FTIR, ^1^H NMR and ^13^C NMR are recorded in Bruker ATR FTIR Alpha II and 400 MHz Bruker spectrophotometer using CDCl_3_ as solvent respectively. Mass spectroscopy is carried out by Thermo Scientific LC–MS with Dionex Ultimate 3000 liquid chromatograph hyphenated with an LTQ XL linear ion trap mass spectrometer. Leica DM2700P polarizing optical microscope (POM) equipped with a Linkam hot stage controller is used for the phase characterization of the synthesized liquid crystals and thermal characterization is carried out by Differential Scanning Calorimetry 6000 PerkinElmer with 10 ^o^C/min scans. The DFT calculations are carried out using Gaussian 09 software. The liquid crystalline compound is optimized by using DFT with the conventional basis set 6-311G (d, p) paired with (B3LYP) hybrid functional.

### Synthesis of LCs

The liquid crystals of the present work are synthesized by following the reported procedure^26^.

#### Step 1: Synthesis of 4-((2′, 4′-difluorophenylimino)methyl) phenol [X]

**Scheme 1:**
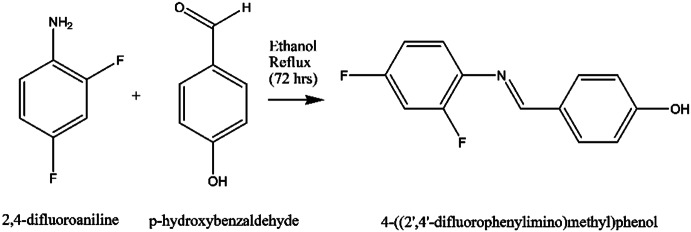
Synthesis of the compound, X.

An equal amount of 2,4-difluoroaniline (1.0 mmol) and p-hydroxybenzaldehyde (1.0 mmol) is taken and refluxed in 10 mL of ethanol for 72 h. The reaction mixture is allowed to cool at room temperature and then kept in ice bath for 2 h. The crystallized product is filtered and recrystallized with ethanol (Scheme 1).

#### Step 2: Synthesis of 4-((2′,4′-diflourophenylimino)methyl)phenyl-4″-pentyloxybenzoate, (5OFB)

**Scheme 2:**
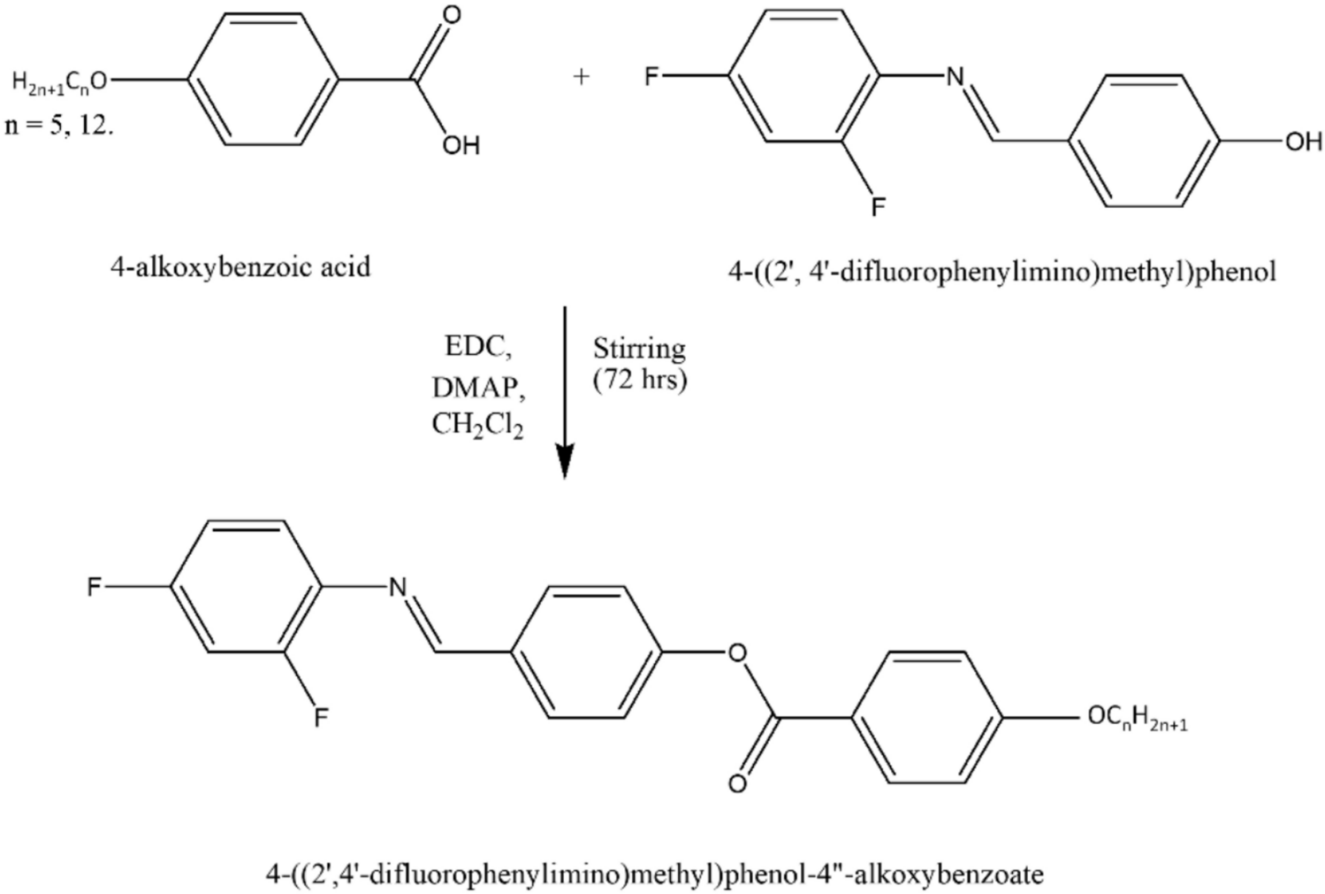
Synthesis of the compounds 5OFB, 12OFB.

Compound X and 4-pentyloxybenzoic acid (0.01 mol) are taken in equal proportion and dissolved in dry methylene chloride (DCM) (30 mL). To the reaction mixture 0.02 mol N-(3-Dimethylaminopropyl)-N′-ethyl carbodiimide hydrochloride (EDC) and small amount of 4-dimethylaminopyridine (DMAP) are added. The reaction mixture is stirred for 72 h at room temperature. Once stirring is done the dicyclohexylurea (DCU) byproduct is obtained which is separated. The filtrate is evaporated, and the obtained product is recrystallized from ethanol (Scheme 2).

The compound, 4-((2′,4′-diflourophenylimino)methyl)phenyl-4′′-dodecyloxybenzoate, (12OFB) is similarly synthesized.

## Results and discussion

### Characterization by ^1^H NMR and FTIR

4-((2′,4′-diflourophenylimino)methyl)phenyl-4′′-pentyloxybenzoate, (5OFB): white (85%); FTIR (cm^−1^): 1722 (C = O *str.*), 1600 (C = N *str.*), 1200 (C–F *str.*) (Fig. SF1); ^1^H NMR (400 MHz, CDCl_3_, ppm): δ 8.45 (s, 1H) δ 8.09–8.06 (d, 2H, J = 8.8 Hz), δ 7.92–7.90 (d, 2H, J = 8.4 Hz) δ 7.28–7.26 (d, 2H, J = 8.4 Hz), δ 7.34–7.05 (m, 3H), 6.92–6.81 (m, 4H) δ 3.99–3.96 (t, 2H) δ 1.8–0.78 (m, 9H ) δ 1.6 (H_2_O present in CDCl_3_) (Fig. SF2). ^13^C NMR (75 MHz, CDCl_3_, ppm): δ 163.55, 162.71, 160.62, 152.84, 132.40, 131.42, 131.35, 130.23, 129.16, 121.60, 121.33, 120.08, 113.35, 110.21, 103.66, 67.33, 28.68, 27.76, 27.10, 21.41, 12.99 (Fig. SF3); MS: m/z 424 MH^+^ (Fig. SF4).

4-((2′,4′-diflourophenylimino)methyl)phenyl-4′′-dodecyloxybenzoate, (12OFB): white (85%) 4; FTIR (cm^−1^): 1730 (C=O *str.*), 1598 (C=N *str.*), 1204 (C–F *str.*) (Fig. SF5); ^1^H NMR (400 MHz, CDCl_3_, ppm): δ 8.45 (s, 1H) δ 8.09–8.07 (d, 2H, J = 8.8 Hz), δ 7.92–7.90 (d, 2H, J = 8.4 Hz) δ 7.28–7.26 (d, 2H, J = 8.4 Hz), δ 6.92–6.90 (d, 2H, J = 8.4 Hz), 6.88–6.80 (m, 4H) δ 3.99–3.96 (t, 2H) δ 1.79–0.77 (m, 23H ) δ 1.6 (H_2_O present in CDCl_3_) (Fig. SF6). ^13^C NMR (75 MHz, CDCl_3_, ppm): δ 163.54, 162.72, 160.61, 152.84, 132.40, 131.35, 129.16, 121.59, 121.33, 120.08, 113.35, 113.00, 110.42, 103.67, 67.35, 30.90, 28.68, 28.63, 28.61, 28.57, 28.53, 28.33, 28.06, 24.95, 21.67, 13.10 (Fig. SF7); MS: m/z 305 (M-C_19_O_3_H_29_)^+^ (Fig. SF8).

### Phase characterization by POM

**Fig. 1:**
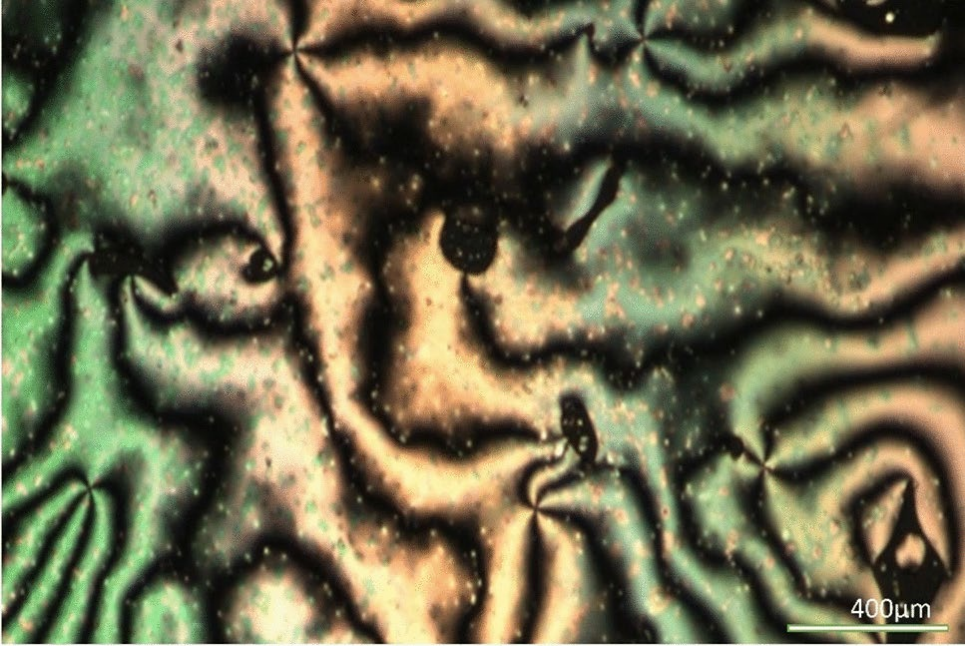
Schlieren texture of 5OFB at 140 ^o^C (10X).

**Fig. 2:**
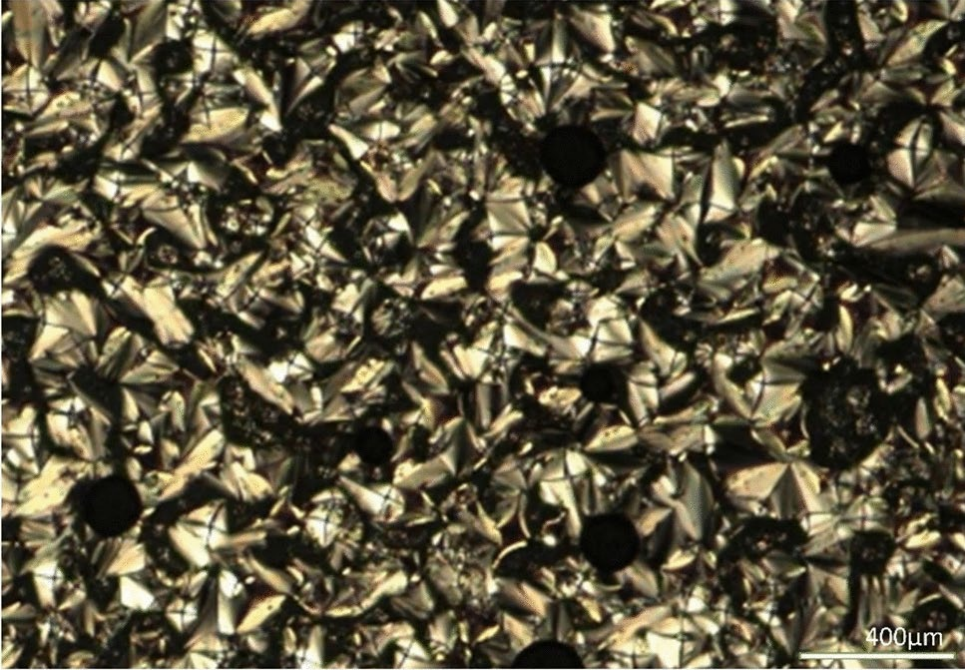
Focal conic fans of 12OFB at 105 ^o^C (10X).

From the POM studies it is observed that the liquid crystal, 5OFB is exhibiting nematic phase by developing schlieren textures during heating and cooling cycles (Fig. 1) while 12OFB exhibited focal conic fans indicating the Smectic A phase (Fig. 2).

### Thermal characterization by DSC

**Fig. 3:**
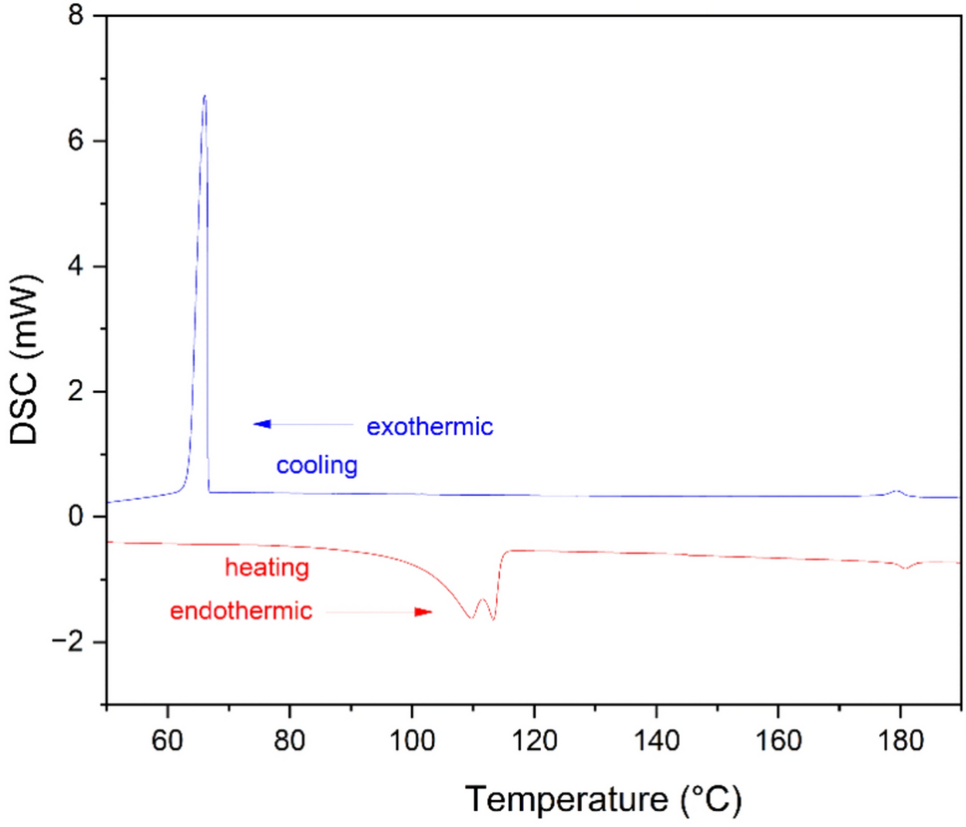
DSC thermograms of 5OFB.

The DSC thermograms are obtained by heating and cooling the sample in a sealed aluminium pan at a rate of 10 ^o^C/min. The data is reported for the second run of the sample. It is observed that the results are reproducible. On heating, 5OFB transformed into isotropic liquid at 180.81 ^o^C with an enthalpy of 0.76 J/g and on cooling transformed into nematic phase at 179.29 ^o^C with an enthalpy change of 0.97 J/g and on further cooling transformed into crystal at 65.98 ^o^C with an enthalpy change of 44.16 J/g (Fig. 3).

**Fig. 4:**
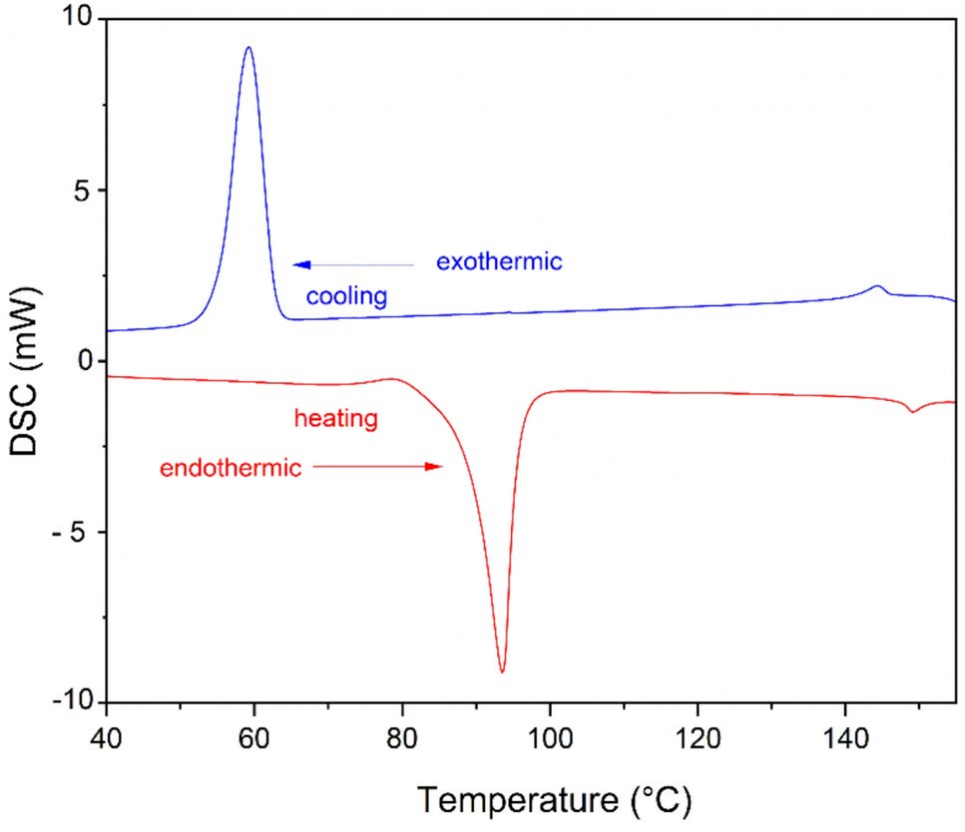
DSC thermograms of 12OFB.

On heating, the liquid crystal, 12OFB on heating transformed into isotropic liquid at 149.55 ^o^C with an enthalpy of 0.71 J/g and on cooling transformed into smectic A phase at 146.02 ^o^C with an enthalpy change of 1.21 J/g and on further cooling transformed into crystal at 59.32 ^o^C with an enthalpy change of 70.04 J/g (Fig. 4). The DSC data is presented in (Table 1).

**Table 1:** Transition temperatures (^o^C) and enthalpy changes (J/g) of 5OFB and 12OFB.

Compound	Phase change	Transition temperature (^o^C)	Enthalpy change (J/g)
5OFB (heating cycle)	Cryst-N	113.3	36.07
	N-Iso	180.80	0.76
5OFB (cooling cycle)	Iso-N	179.29	0.97
	N-Cryst	65.98	44.16
12OFB (heating cycle)	Cryst-SmA	95.14	67.29
	SmA-Iso	149.65	0.71
12OFB (cooling cycle)	Iso-SmA	146.02	1.21
	SmA-Cryst	59.32	70.04

In the present work, the fluorine substituents in the terminal and lateral positions have been found to enhance the mesomorphic thermal stabilities. The mesomorphic behaviour of these compounds are compared with the structurally similar compound reported by M Hagar et. al^27^. In their work, a new liquid crystal Schiff base possessing ester linkage is obtained where the aniline used is without any substitution. In our present work, 2,4-difluoro aniline is used for the preparation of liquid crystals. It is observed that the lower chain length compounds without fluorine exhibited a lower mesomorphic range of 46^o^ as compared to the compounds of the present work which are exhibiting a mesomorphism range of 113.3^o^. Similarly, in the higher chain length members, the compound without fluorine exhibits liquid crystalline behaviour for around 33^o^ while the compound of the present study is exhibiting mesomorphism for a range of 86.7^o^. This is a clear indication of fluorine influencing mesomorphism. Fluorine substituents are influencing the mesomorphism by enhancing the dipole moment of the molecules. On comparing the compounds of the present work with pure alkoxy benzoic acids (nOBAs), it is observed that there is enhancement in the mesomorphic range of the nematic phase as compared to nOBAs (Table 2). The high melting temperature of the nOBAs may be due to their dimeric nature through hydrogen bonding interactions and has more attractive forces between the molecules in its crystal lattice. When these acids are involved in the formation of esters, the dimeric nature of the acids are lost leading to new interactions with the phenol counterpart. This leads to the enhancement of the polarity or polarizability of the newly formed esters, leading to the enhancement in the mesogenic behaviour. It is observed that as the chain length of the alkoxy chains increases from pentyloxy to dodecyloxy, the nematic mesophase is curbed and only smectic A mesophase is exhibited which is attributed to the intertwining of long alkyl chains.

**Table 2:** Comparison of nOBAs with 5OFB and 12OFB.

Compound	Phase variant	(ΔT)_nematic_	(ΔT)_smectic_	(ΔT)_LC_
5OBA	N	30.0	–	30.0
5OFB	N	113.3	–	113.3
12OBA	N, SmC	5.0	37.0	42.0
12OFB	SmA	–	86.7	86.7

### Computational details

Gaussian 09 software is used to carry out the DFT calculations. Optimization of 5OFB is carried out with the conventional basis set 6-311G (d, p) paired with B3LYP hybrid functional for the correlation component and the Becke three hybrid density functionals for the exchange part^28–31^.

#### Nonlinear optical properties (NLO)

**Fig. 5:**
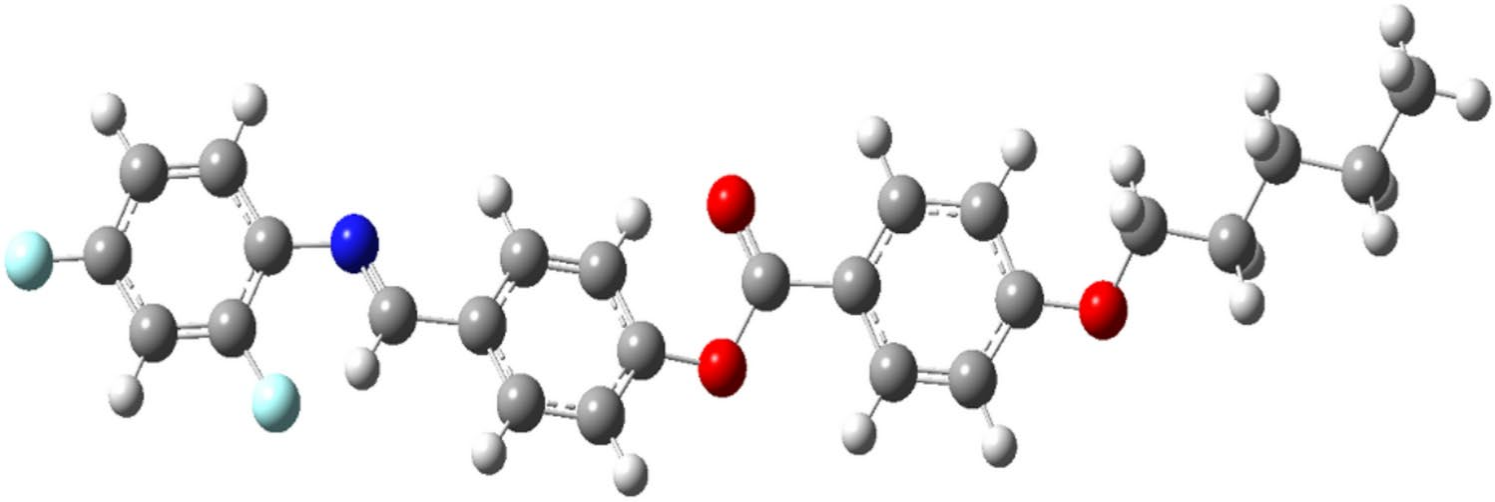
Optimized structure of 5OFB.

The long molecular chains and the ability of the molecules to respond to electric fields make these liquid crystals show strong nonlinear optical effects. The liquid crystal, 5OFB is optimized (Fig. 5) and the total polarizability, total dipole moment, anisotropic polarizability, asymmetry parameters and first order hyperpolarizability is calculated (Table 3).

**Table 3:** Nonlinear optical (NLO) properties of 5OFB.

Dipole moment (μ) (in debye)				
Compound	μ_X_	μ_Y_	μ_Z_	μ_total_
5OFB	2.1497914	− 0.0422435	0.2183842	2.1612679
Polatizability (in debye-Å)				
α_XX_	612.1646009			
α_YY_	249.9519916			
α_ZZ_	171.8550928			
α^iso^	344.6572284			
Δα	401.2610587			
ŋ	1.645971489			
Hyperpolarizability (in debye-Å^2^)				
β_XXX_	−2433.9686533			
β_YYY_	62.7716353			
β_ZZZ_	27.2114179			
β_XXY_	280.7060646			
β_XYY_	129.3978128			
β_XXZ_	254.2060405			
β_XYZ_	−7.0866363			
β_YYZ_	−3.1965921			
β_XZZ_	110.2126814			
β_YZZ_	140808032			
β_0_	140808375.5			

Table 3 displays the components of polarizability and hyperpolarizability tensors such as anisotropic polarizability (Δα), isotropic polarizability (α^iso)^, asymmetric parameters(ƞ), total polarizability (α_total_), total molecular dipole moment (μ_total_), first order hyperpolarizability (β_0_). It is observed that components of dipole moment, polarizability, and hyperpolarizability have greater magnitudes which is justified by the compound’s smaller energy gaps^32–34^. The NLO characteristics of the molecules are found to be enhanced. The formulas used to find NLO characteristics are as follows.

1 $$\mu { }_{total}=\sqrt{{{\mu }_{x}}^{2}+{{\mu }_{y}}^{2}+{{\mu }_{z}}^{2}}$$

2 $${\alpha }_{total =}\frac{1}{\sqrt{2}}\sqrt[]{{\left({\alpha }_{xx}-{\alpha }_{xy}\right)}^{2}+{\left({\alpha }_{yy}-{\alpha }_{zz}\right)}^{2}+{\left({\alpha }_{zz}-{\alpha }_{xx}\right)}^{2}+6{\alpha }_{xy}^{2}+6{\alpha }_{xz}^{2}+6{\alpha }_{yz}^{2}}$$

3 $$\eta =\frac{{\alpha }_{xx}-{\alpha }_{zz}}{{\alpha }_{xx}-{\alpha }_{iso}}$$

4 $$\Delta \alpha ={\alpha }_{xx}-\frac{{\alpha }_{yy}+{\alpha }_{zz}}{2}$$

5 $${\alpha }^{iso}=\frac{{\alpha }_{xx}+{\alpha }_{yy}+{\alpha }_{zz}}{3}$$

6 $${\beta }_{x}={\beta }_{xxx}+{\beta }_{xyy}+{\beta }_{xzz}$$

7 $${\beta }_{y}={\beta }_{yyy}+{\beta }_{xxy}+{\beta }_{yzz}$$

8 $${\beta }_{z}={\beta }_{zzz}+{\beta }_{xxz}+{\beta }_{yyz}$$

9 $${\beta }_{0}=\sqrt{{{\beta }_{x}}^{2}+{{\beta }_{y}}^{2}+{{\beta }_{z}}^{2}}$$

#### Electrostatic potential (ESP)

**Fig. 6:**
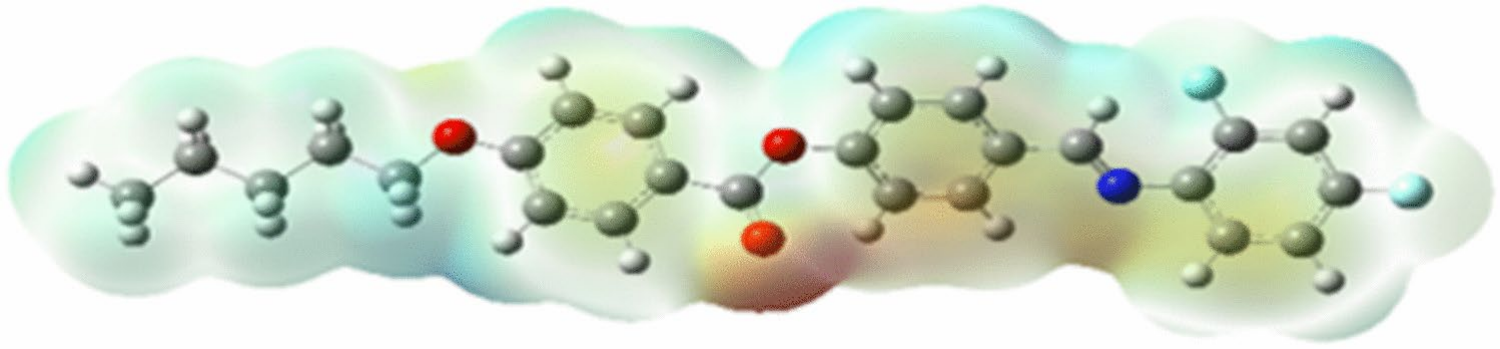
ESP of 5OFB.

Molecular behavior, structural conformation and electrostatic potential distribution are carried out through DFT studies. The electrostatic potential surface shown in (Fig. 6) describes the molecule’s electrophilic and nucleophilic characteristics. The largest amount of the positive zone, where the nucleophilic reaction occurs, is shown by the light blue color in the ESP graphs, whereas the negative region, where the electrophilic reaction occurs, is represented by the reddish region. The dull reddish colour is an indication of the low electrostatic potential of the compound’s fluorinated terminal chain. The compound’s greatest electronegativity potential is represented by the dark red color in the picture at N and O atoms. The sky-blue color at the terminal alkyl chain and core symbolizes the compound’s positive electrostatic potential.

The total ESP analysis shows the homogeneously spread electron density throughout the compound. The compound’s electrostatic potential surface indicated by different colors imply the potential increase in the order of red, orange, yellow, green, and blue.

#### HOMO-LUMO and reactivity parameters

**Fig. 7:**
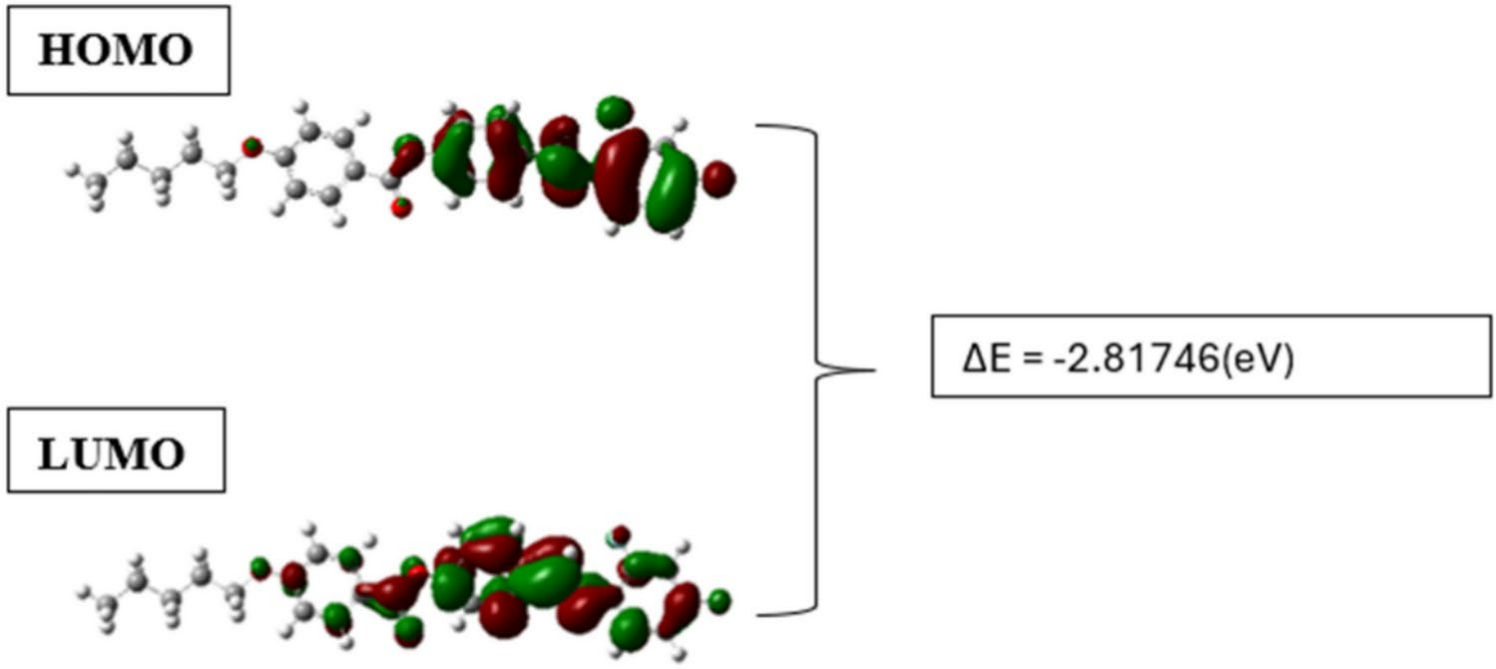
HOMO–LUMO of 5OFB.

Studies on the highest occupied molecular orbitals (HOMO), lowest unoccupied molecular orbitals (LUMO) and the energy gap helps understand the optical, electrical properties, stability and reactivity of liquid crystals (Fig. 7). A large energy gap is an indication of low reactivity while a low energy gap indicates better contact with other molecules and polarization. A reduced energy gap in the molecule of this work indicates unusual optoelectrical behavior due to the easily polarizing nature^32,33,35–37^.

The HOMO-LUMO calculations, energy gaps and reactivity parameters of the optimized molecule are calculated based on the following formulas.

10 $$\upmu = \frac{-I+A}{2}$$

11 $$\eta =\frac{I-A}{2}$$

12 $$\upomega = \frac{{\upmu }^{2}}{2\eta }$$

13 $$I= -{E}_{HOMO}$$

14 $$A= -{E}_{LUMO}$$

15 $$S=\frac{1}{\eta }$$

16 $$\chi =-\mu$$

The chemical reactivity parameters like Ionization potential (I), Electron-affinity (A), Electronegativity (χ), Chemical hardness (ƞ), Global Softness (S), Chemical potential (µ), electrophilicity index(ω), calculated for 5OFB is presented in (Table 4).

**Table 4:** Chemical reactivity of 5OFB.

Reactivity parameters	
E_HOMO_ (in hartree)	−0.30055
E_LUMA_ (in hartree)	−0.19701
Ionization potential (I) (in hartree)	0.30055
Electron-affinity (A) (in hartree)	0.19701
Electronegativity (χ) (in hartree)	0.05177
Global hardness (η) (in hartree^−1^)	0.05177
Global softness (s) (in hartree)	19.31621
Chemical potential (μ) (in hartree)	−0.05177
Global electrophilicity index (ω) (in hartree)	0.025885

## Conclusion

Fluorine containing liquid crystals exhibiting nematic and smectic A phase are successfully synthesized. The versatile nature of the fluorine atom in possessing high electronegativity with small size are responsible for the liquid crystal molecules in possessing these outstanding features. The length of the terminal alkoxy chain is influencing the mesomorphism with shorter chains favoring the nematic phase while the longer chains are inducing smectic A phase. A wide range of mesomorphism is realized in comparison to pure alkoxy benzoic acids. Azomethine (–CH=N–) linkage increases the polarizability of the molecule and consequently enhances the intermolecular association between the molecules leading to the realization of mesomorphism. Nonlinear optical properties of the compound demonstrated the suitability of applications of these molecules in electronic devices. The alkoxy chain length-driven tunability of the mesophase transitions, in conjunction with the effects of fluorine substitution and Schiff base linkage, opens new possibilities for the creation of sophisticated materials for application in photonics and display technologies.

## Supplementary Information

Supplementary Information.

## Data Availability

The authors declare that the data supporting the findings of this study are available within the paper and its Supplementary Information files.
