# Supplementary material for: Mesomorphic and DFT study of new difluoro substituted Schiff base liquid crystals
Source: Sci Rep. 2025 May 3;15:15540. doi: 10.1038/s41598-025-97375-8 (PMC12049477; doi:10.1038/s41598-025-97375-8)
Supplement: Supplementary file 1 — Supplementary Information. [file 41598_2025_97375_MOESM1_ESM.docx]

Supplementary Information

for

**Synthesis of New Difluoro-substituted Schiff Base Ester Liquid Crystals: Mesomorphism and DFT study**

Manav Jeetendra Shirodkar^1^, K Subrahmanya Bhat^1^, Debanjan Bhattacharjee^2^, Sonali M K^1^, Mahesha M G^3^, Poornima Bhagavath^1*^

^1^Department of Chemistry, Manipal Institute of Technology, Manipal Academy of Higher Education, Manipal -576 104, Karnataka, India

^2^Department of Physics, Manipal University Jaipur, Jaipur, India

^3^Department of Physics, Manipal Institute of Technology, Manipal Academy of Higher Education, Manipal -576 104, Karnataka, India

| Contents | | |
| --- | --- | --- |
| Experimental Details: Figures Page No | | |
| Figure SF1 | FTIR spectrum of 5OFB | 4 |
| Figure SF2 | ^1^H NMR spectrum of 5OFB | 4 |
| Figure SF3 | ^13^C NMR spectrum of 5OFB | 4 |
| Figure SF4 | Mass spectra of 5OFB | 4 |
| Figure SF5 | FTIR spectrum of 12OFB | 4 |
| Figure SF6 | ^1^H NMR spectrum of 12OFB | 4 |
| Figure SF7 | ^13^C NMR spectrum of 12OFB | 4 |
| Figure SF8 | Mass spectra of 12OFB | 4 |


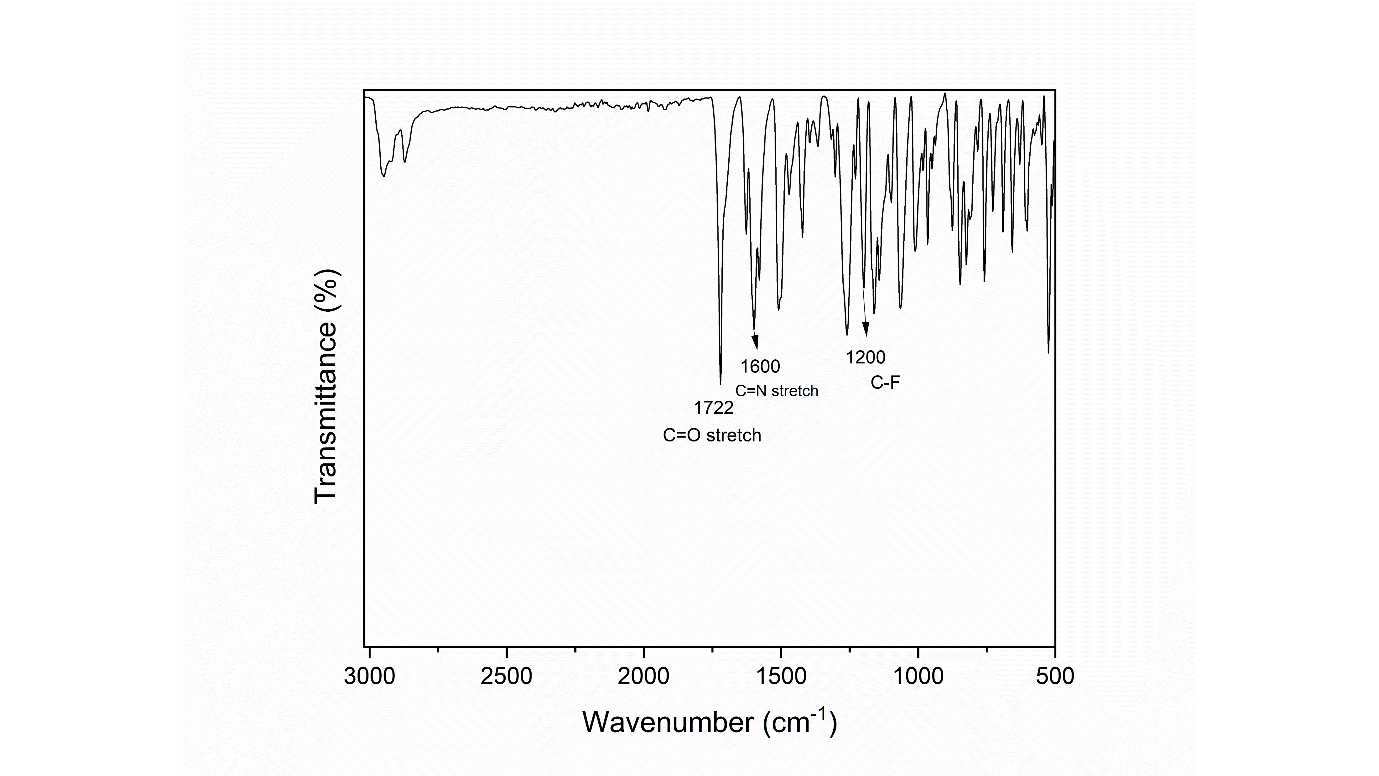


**Figure SF1: FTIR spectrum of 5OFB**


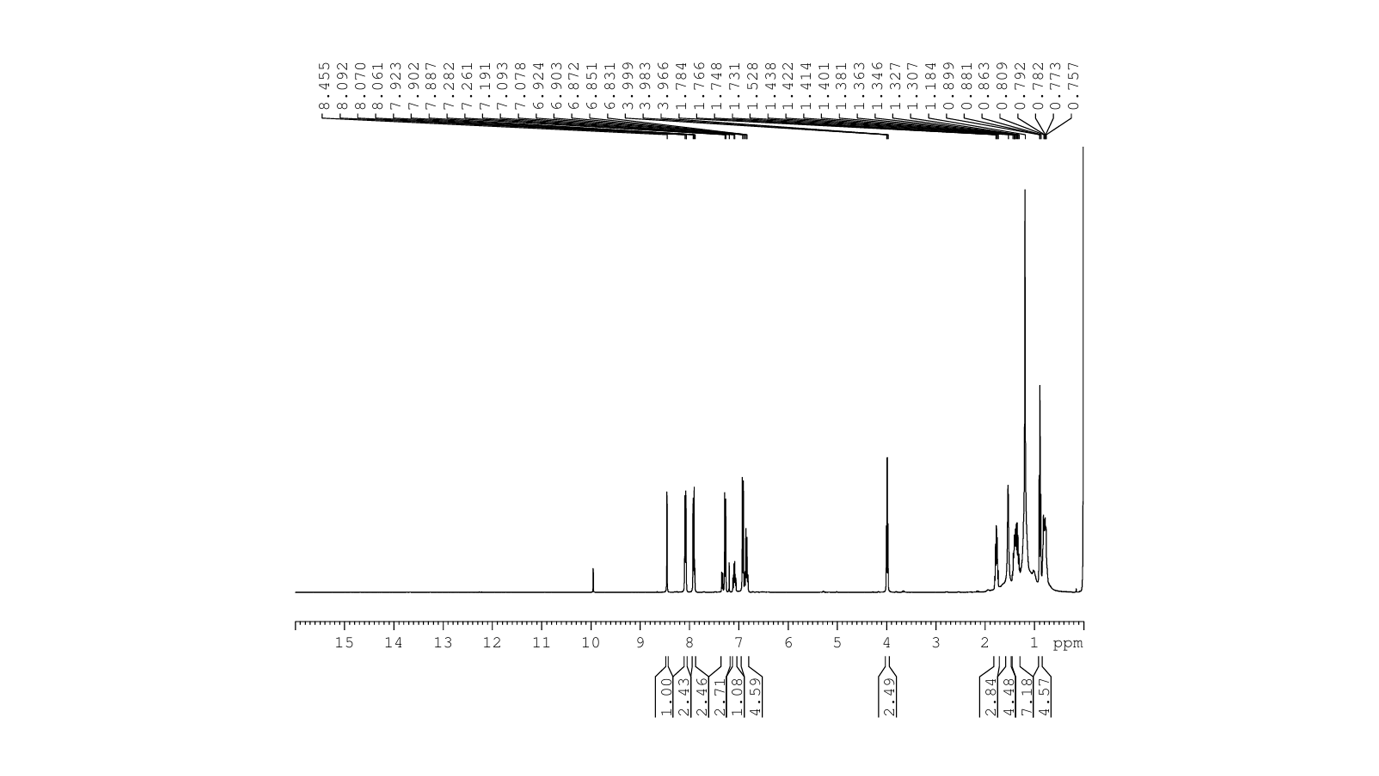


**Figure SF2: ^1^H NMR spectrum of 5OFB**

**
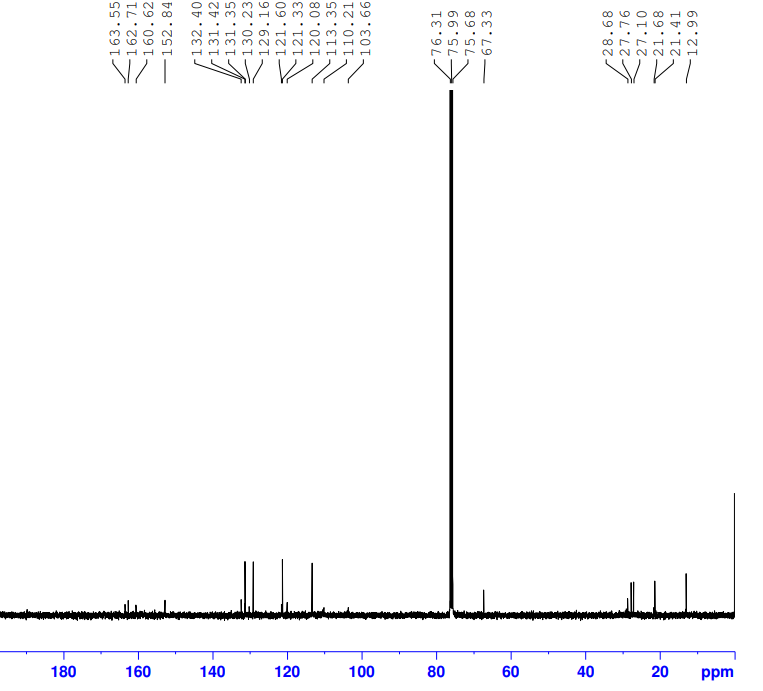
**

**Figure SF3: ^13^C NMR spectrum of 5OFB**

**
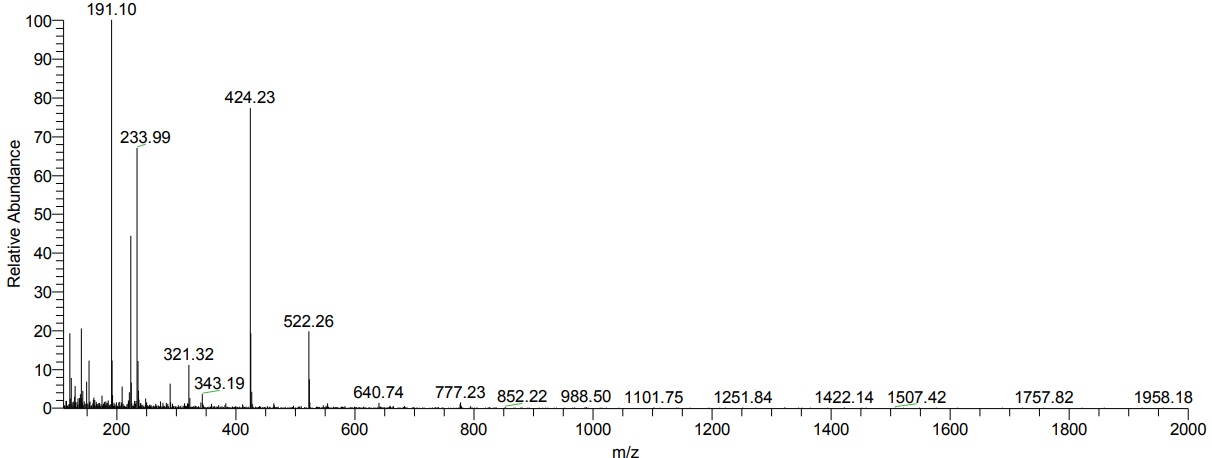
Figure SF4: Mass spectrum of 5OFB**


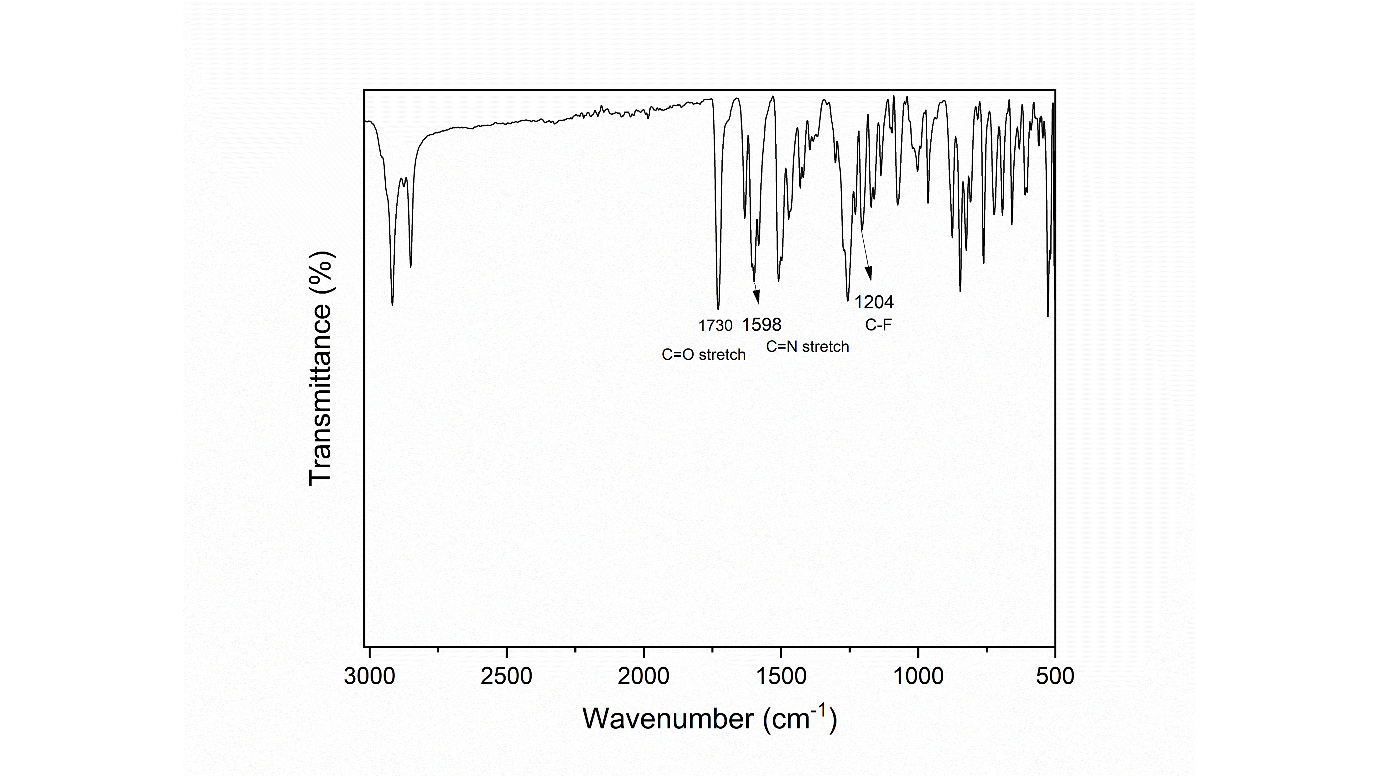


**Figure SF5: FTIR spectrum of 12OFB**


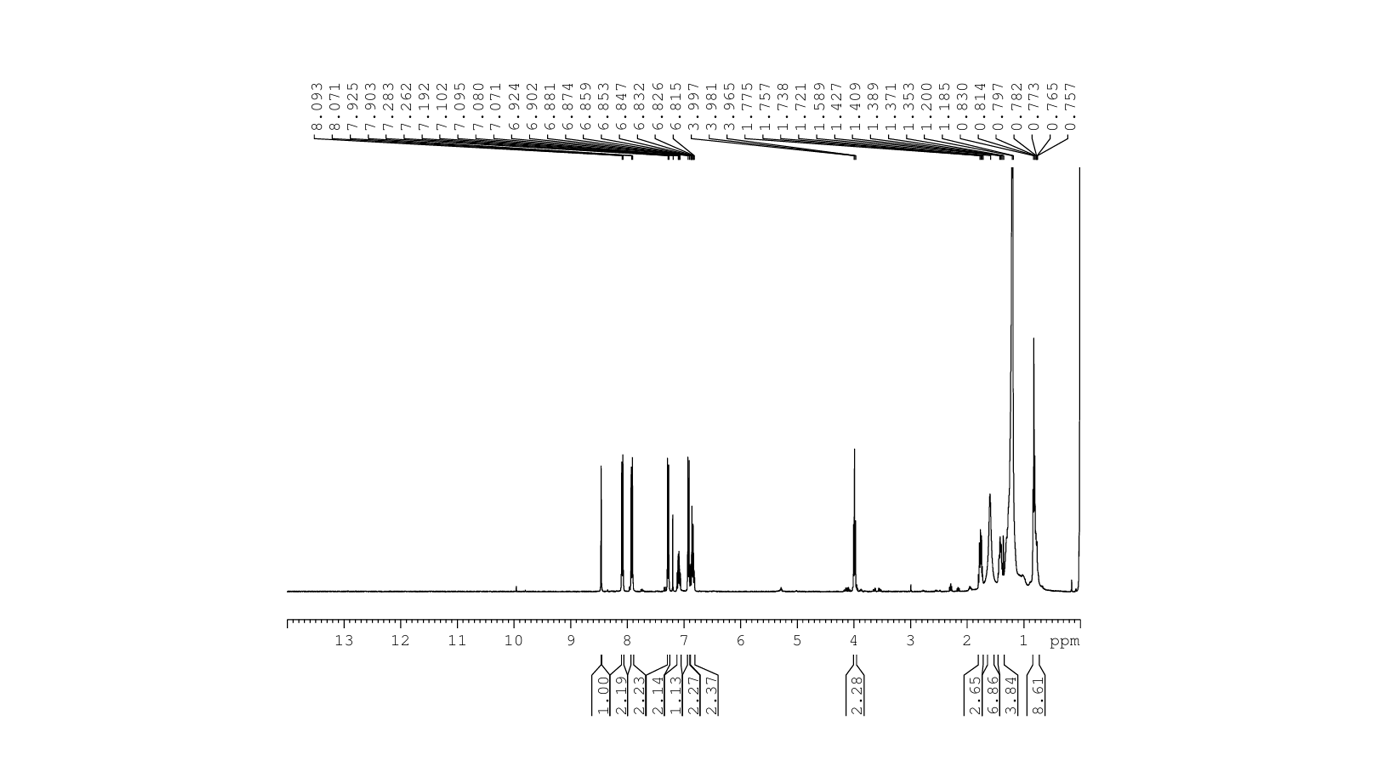


**Figure SF6: ^1^H NMR spectrum of 12OFB**

**
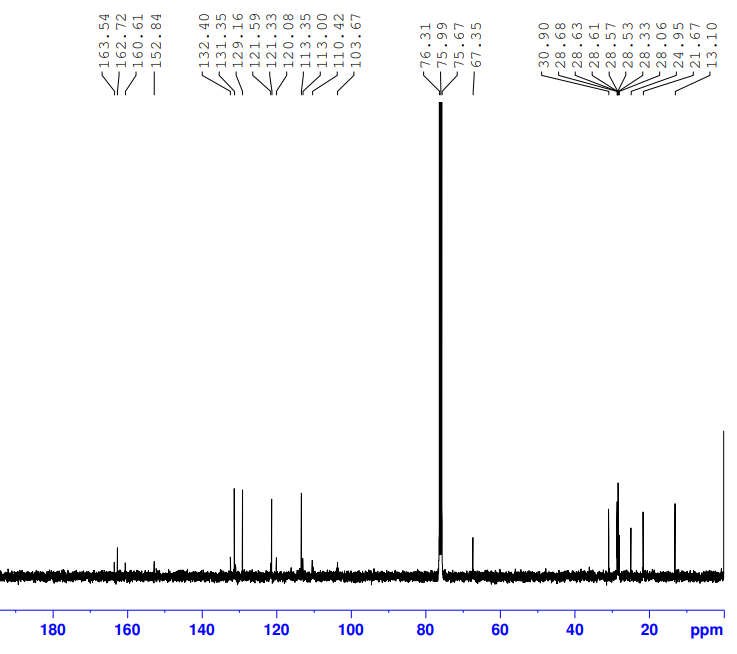
**

**Figure SF7: ^13^C NMR spectrum of 12OFB**

**
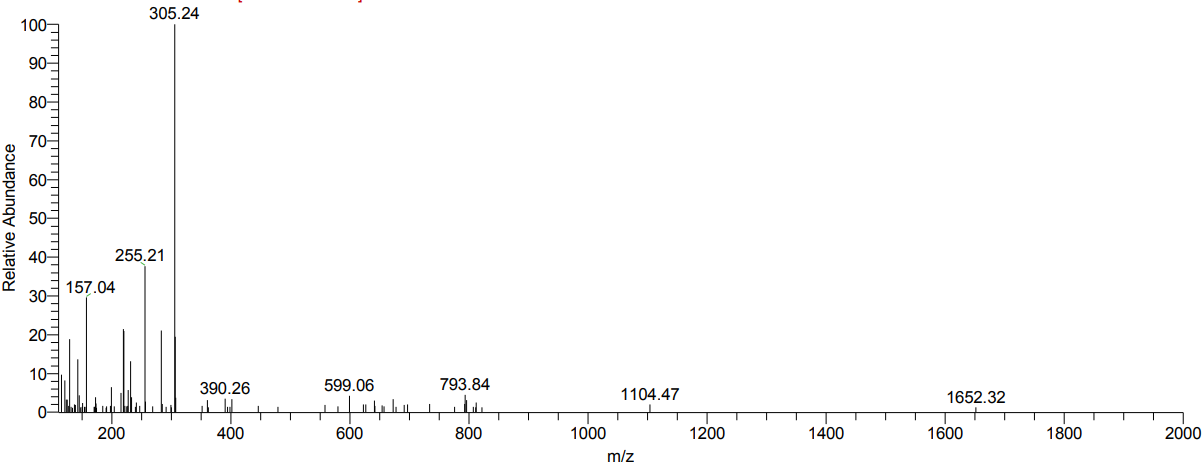
Figure SF8: Mass spectrum of 12OFB**
